# Combined Association of High-Sensitivity C-Reactive Protein and Vitamin D with Short-Term Functional Outcomes in Acute Ischemic Stroke

**DOI:** 10.3390/nu18193244

**Published:** 2026-10-01

**Authors:** Seongmin Choi, Yunjung Rho, Min-Su Kim, Myung Chul Yoo, Ga Yang Shim, Jin San Lee, Sung Joon Chung, Yunsoo Soh

**Affiliations:** 1 Department of Physical Medicine & Rehabilitation, Kyung Hee University Hospital, College of Medicine, Kyung Hee University, Seoul 02447, Republic of Korea; 15224@khmc.or.kr (S.C.);; 2 Department of Neurology, Kyung Hee University Hospital, College of Medicine, Kyung Hee University, Seoul 02447, Republic of Korea; 3 Department of Physical Medicine & Rehabilitation, Kyung Hee University Hospital at Gangdong, College of Medicine, Kyung Hee University, Seoul 05278, Republic of Korea

**Keywords:** high-sensitivity C-reactive protein, vitamin D deficiency, acute ischemic stroke, inflammation, nutritional status, functional outcome, rehabilitation

## Abstract

**Background/Objectives**: Whether high-sensitivity C-reactive protein (hsCRP) and vitamin D (Vit D) provide complementary prognostic information for acute ischemic stroke is unclear. We examined the separate and joint associations of serum hsCRP and Vit D levels with short-term functional outcomes after acute ischemic stroke. **Methods**: We retrospectively analyzed 192 patients with brain magnetic resonance imaging-confirmed acute ischemic stroke admitted to a single tertiary center. Stroke severity at admission was graded using the National Institutes of Health Stroke Scale. Physical and cognitive recovery were assessed using the Berg Balance Scale (BBS), Manual Function Test (MFT), Korean Mini-Mental State Examination (K-MMSE), and Korean version of the modified Barthel Index, while disability was graded using the modified Rankin Scale (mRS) at discharge. **Results**: Among 192 patients stratified into low- (<1.0 mg/L, *n* = 76), moderate- (1.0–3.0 mg/L, *n* = 57), and high- (>3.0 mg/L, *n* = 59) hsCRP groups, serum 25-hydroxyvitamin D fell stepwise as hsCRP rose, and high hsCRP was independently associated with poorer BBS, MFT, K-MMSE, and mRS outcomes. Patients with both high hsCRP and Vit D deficiency (<20 ng/mL) formed the highest-risk group (adjusted odds ratio up to 12.54), with the risk increasing stepwise as the number of adverse markers increased. No significant multiplicative interaction was observed, whereas a significant additive interaction was found only for cognitive impairment. **Conclusions**: Patients with both elevated hsCRP and Vit D deficiency showed the highest odds of poor short-term outcomes. A simple count of these adverse markers may help identify patients at higher risk after acute ischemic stroke and may help define a subgroup for further evaluation in prospective intervention trials of Vit D supplementation.

## 1. Introduction

Acute ischemic stroke frequently leaves survivors with lasting motor, cognitive, and functional impairments, and recovery over the first weeks varies widely between individuals [1,2]. Identifying patients at the bedside who are likely to recover poorly would help target rehabilitation resources [3]. As routine admission blood tests are inexpensive, reproducible, and universally available, biomarkers derived from them are attractive candidates for early prognostication, and a growing body of research has linked these markers to post-stroke severity and recovery [4,5].

Inflammation is the principal driver of secondary injury after cerebral ischemia, and several inflammation-related blood indices are associated with worse outcomes. Our group previously showed that a higher neutrophil-to-lymphocyte ratio, a leukocyte-based index of systemic inflammation, predicts poorer short-term cognitive and daily living outcomes in patients with acute ischemic stroke [6]. High-sensitivity C-reactive protein (hsCRP) is a pentraxin acute-phase protein synthesized by hepatocytes in response to interleukin-6 (IL-6) [7]. Because hsCRP is stable in serum, inexpensive, and quantifiable by standardized high-sensitivity assays in the low milligram-per-liter range, it has become the most widely used clinical index of low-grade systemic inflammation and a guideline-endorsed marker of cardiovascular risk [8,9]. Its prognostic reach extends across cerebrovascular disease. Meta-analytic data linked higher hsCRP to greater stroke severity and unfavorable functional outcomes [10], and large prospective cohorts showed that hsCRP predicts functional disability and stroke recurrence after acute ischemic stroke, an association mediated only in part by initial severity [11]. Beyond the vasculature, hsCRP also tracks nutritional and musculoskeletal status, and higher hsCRP levels are associated with low muscle strength in Korean adults [12]. Additionally, muscle mass deficit at admission predicts greater stroke severity and worse discharge outcome [13], underscoring how tightly systemic inflammation, nutritional reserve, and physical recovery are interwoven. Therefore, hsCRP is best regarded not as an inert bystander but as a readout of an inflammatory milieu that plausibly shapes recovery, supporting the broader premise that admission blood chemistry alone can meaningfully inform short-term prognosis.

Vitamin D (Vit D) is a nutritional counterweight for this inflammatory response. Vit D deficiency is among the most prevalent nutritional disorders worldwide and is especially common in older adults, hospitalized populations, and East Asian countries, reflecting limited sun exposure, low dietary intake, and reduced cutaneous synthesis with age [14], which is precisely the profile of many patients presenting with acute stroke. Mechanistically, Vit D directly counter-regulates the pathway that generates elevated hsCRP levels. Immune and neural cells express both the Vit D receptor and the enzymes that activate Vit D [15]. Moreover, 1,25-dihydroxyvitamin D suppresses nuclear factor-κB signaling and downregulates IL-6 and tumor necrosis factor-α, with IL-6 being the principal hepatic stimulus for CRP synthesis, while promoting anti-inflammatory interleukin-10 and steering microglia toward a less inflammatory phenotype after ischemic injury [16]. Critically, the link between the two markers is not merely parallel but appears causal and directional. Clinical studies have reported an inverse association between serum 25-hydroxyvitamin D (25-OHD) and circulating CRP in inflammatory diseases and acute ischemic stroke [17,18]. Bidirectional Mendelian randomization indicates that low Vit D drives higher CRP rather than the reverse, with the effect confined to the deficiency range [19]. This restriction is pivotal for the present hypothesis: if Vit D limits CRP production particularly when Vit D is deficient, then a nutritionally deficient patient has effectively lost this brake, so that a concurrently high hsCRP level signals an inflammatory response that is at once amplified and inadequately opposed. Therefore, we reasoned that elevated hsCRP and Vit D deficiency should not merely co-occur, but should compound one another’s prognostic signal, and patients with both maximal inflammatory drive and minimal nutritional reserve show the poorest recovery.

Our previous study in this cohort established that Vit D deficiency alone was associated with greater admission severity and poorer short-term recovery, but examined Vit D in isolation [20]. This single-marker framework resulted in two clinically important gaps. First, it could not explain why Vit D-deficient patients fared worse; because Vit D acts largely by restraining inflammation, its prognostic significance should depend on the opposing inflammatory state. Second, it treated all Vit D-deficient patients as a single group; however, a deficient patient with a quiescent inflammatory response is unlikely to carry the same risk as a patient with an active, unopposed response. The present analysis therefore addressed a mechanistically driven hypothesis that the earlier report could not examine. We hypothesized that jointly considering high hsCRP and Vit D deficiency would identify a cumulative biomarker burden associated with progressively poorer outcomes.

Accordingly, we evaluated the independent and combined associations of admission serum hsCRP and Vit D levels with initial stroke severity and short-term physical, cognitive, and functional outcomes and determined whether their joint burden relates to outcome in an additive or synergistic manner.

## 2. Materials and Methods

### 2.1. Study Population and Design

**Figure 1:**
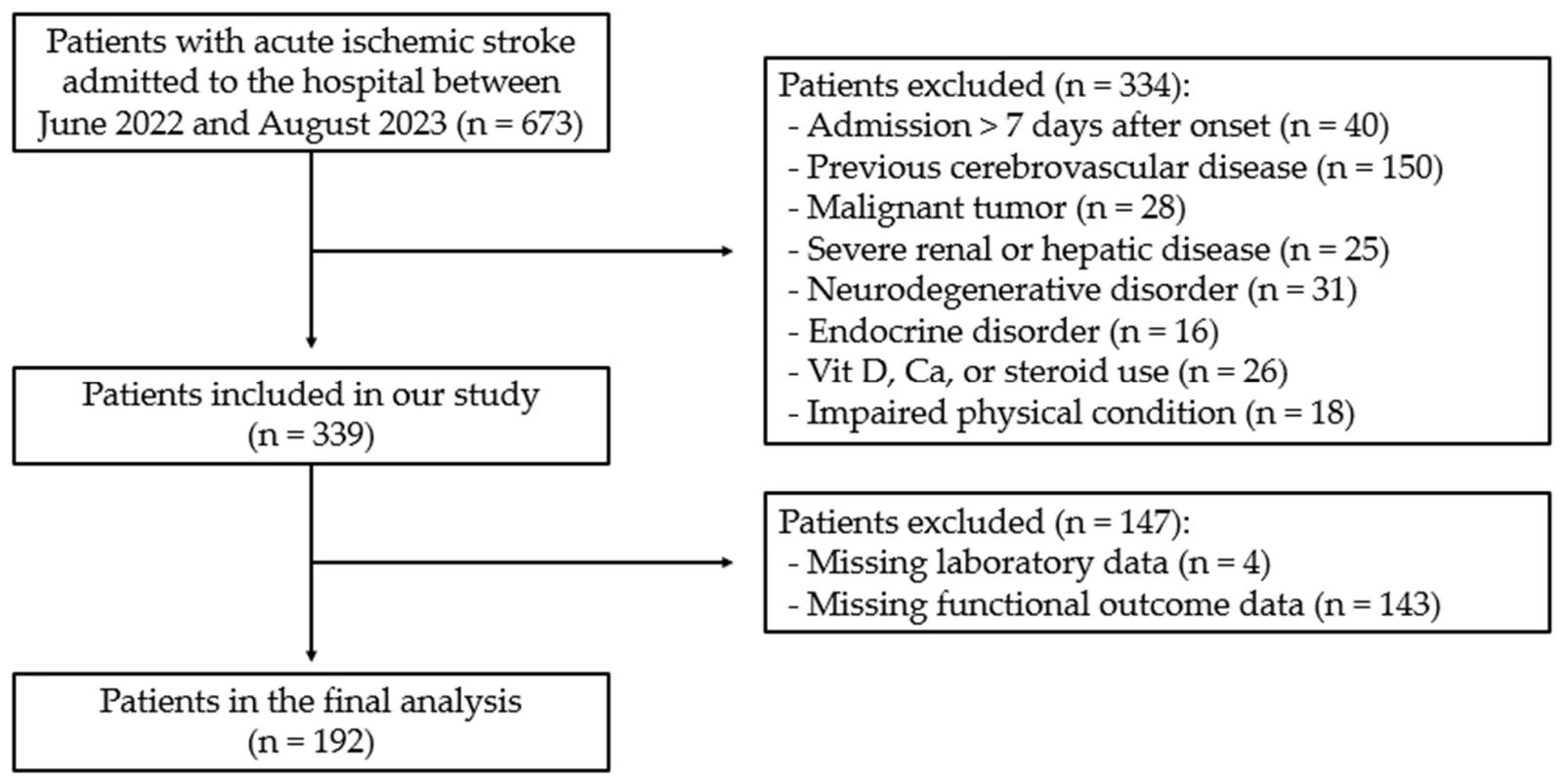
Flow diagram for patient selection.

We conducted a retrospective analysis of consecutive adult patients admitted to Kyung Hee University Hospital (Seoul, Republic of Korea) between June 2022 and August 2023 with brain magnetic resonance imaging-confirmed acute ischemic stroke. The present study was a retrospective secondary analysis investigating the combined and cumulative prognostic value of hsCRP and Vit D, which was not addressed in our previous report [20]. That report modeled Vit D as a single exposure, whereas the present analysis was built on a different exposure structure—the four-way cross-classification of inflammatory and Vit D status and the resulting adverse-marker burden—and deliberately used the same cohort so that the two frameworks could be compared directly without between-cohort confounding. A separate report from an overlapping institutional cohort examined the neutrophil-to-lymphocyte ratio [6]; that analysis used a different exposure and a different biomarker class and evaluated neither Vit D nor any joint-exposure framework. Patients were excluded if they presented more than 7 days post-onset; had a history of cerebrovascular events; had a malignant tumor; suffered from severe renal (stage 4–5 chronic kidney disease or dialysis dependence) or hepatic disease (decompensated liver cirrhosis); carried a neurodegenerative diagnosis (e.g., Parkinsonism or dementia); had thyroid, parathyroid, or adrenal disorders; were taking Vit D, calcium, or corticosteroids; were already functionally dependent (pre-stroke modified Rankin Scale (mRS) ≥ 3); or lacked complete laboratory or functional outcome data. Figure 1 illustrates the selection process. Demographic and clinical data were extracted from the electronic medical records, including age, sex, body mass index, medical history (hypertension, diabetes mellitus, dyslipidemia, and coronary artery disease), smoking status, alcohol consumption, reperfusion treatment (intravenous thrombolysis and endovascular therapy), length of hospital stay, and time from stroke onset to admission. Hypertension, diabetes mellitus, and dyslipidemia were considered present if a corresponding diagnosis had been documented in the medical records prior to the stroke. Participants who smoked more than one cigarette per week were classified as smokers, whereas those who consumed alcohol at least once per week were classified as drinkers. Intravenous thrombolysis was defined as intravenous administration of a thrombolytic agent, whereas endovascular therapy was defined as catheter-based mechanical thrombectomy.

Venous blood samples were collected within 24 h of admission, typically at approximately 6:00 a.m. the following morning. The primary exposures of interest were serum hsCRP (mg/L), indicating systemic inflammation, and total serum 25-OHD (ng/mL), indicating Vit D status. Following established cardiovascular risk thresholds, hsCRP was categorized as low (<1.0 mg/L), moderate (1.0–3.0 mg/L), or high (>3.0 mg/L), and Vit D deficiency was defined as 25-OHD < 20 ng/mL [12,14,21]. For the joint analysis, hsCRP >3.0 mg/L was defined as an adverse marker based on the established high cardiovascular risk threshold, while hsCRP ≤3.0 mg/L was classified as non-high. Patients were assigned to one of four categories (neither adverse marker, high hsCRP only, Vit D deficiency only, or both) and scored according to the count of adverse markers (0, 1, or 2). Additional laboratory variables included complete blood count and serum hemoglobin A1c, total cholesterol, triglyceride, and high- and low-density lipoprotein cholesterol levels. All laboratory measurements were performed at the central laboratory of Kyung Hee University Hospital (Seoul, Republic of Korea). Complete blood counts were measured using Sysmex XN-10/XN-20 analyzers (Sysmex Corporation, Kobe, Japan). Serum hsCRP was measured by latex-enhanced immunoturbidimetry using a Beckman Coulter AU5800 analyzer (Beckman Coulter Inc., Brea, CA, USA), and 25-OHD by a chemiluminescent immunoassay using a UniCel DxI 800 system (Beckman Coulter Inc.). This retrospective study was approved by the Institutional Review Board of Kyung Hee University Hospital (KHUH 2023-10-018), which waived the requirement for informed consent owing to the retrospective study design.

### 2.2. Outcome Measurements

Initial stroke severity upon admission was assessed using the National Institutes of Health Stroke Scale (NIHSS), with scores ≥ 5 denoting moderate-to-severe stroke [13]. Within three weeks of onset, trained therapists and clinicians evaluated short-term recovery using a panel of functional measures. This three-week period was used as the maximum assessment window to maintain a relatively uniform early post-stroke timeframe while accommodating variations in assessment timing in routine clinical practice. The functional measures included the Berg Balance Scale (BBS) for balance (unfavorable outcome, ≤20), the Manual Function Test (MFT) for affected upper limb manual dexterity (unfavorable, ≤19), the Korean Mini-Mental State Examination (K-MMSE) for cognition (unfavorable, ≤23), and the Korean Modified Barthel Index (K-MBI) for activities of daily living (unfavorable, ≤50) [22,23,24,25,26,27]. Global disability at discharge was graded using the mRS, with scores ≥ 3 defined as unfavorable [28]. Throughout this study, we deliberately differentiated the NIHSS score, a baseline measure of initial stroke severity, from the mRS and three-week functional scales, which capture short-term post-stroke recovery.

### 2.3. Statistical Analysis

Data normality was assessed before analysis. Continuous variables were compared across hsCRP groups using one-way analysis of variance or the Kruskal–Wallis test, while categorical variables were evaluated using the chi-square test. Spearman’s rank correlation coefficients were used to describe continuous associations. The association between hsCRP levels and each clinical outcome was evaluated using staged logistic regression. Model 1 was unadjusted; Model 2 was adjusted for age and sex; Model 3 was adjusted for body mass index, hypertension, diabetes mellitus, and 25-OHD; and Model 4 was further adjusted for endovascular therapy. For joint analysis, the four exposure categories (reference: no abnormality) were modeled by adjusting for age, sex and endovascular therapy. The interaction between the two biomarkers was assessed on both multiplicative and additive scales. On the multiplicative scale, interaction was evaluated using the odds ratio for the product term. On the additive scale, the relative excess risk due to interaction (RERI) and the attributable proportion due to interaction (AP) were calculated. Confidence intervals for RERI and AP were estimated using percentile bootstrap with 2000 resamples. To account for multiple comparisons in the logistic regression analyses, the Benjamini–Hochberg false discovery rate correction was applied separately within each test family. Bonferroni-adjusted *p* values were additionally calculated as a more conservative reference. The incremental discriminative value of integrating Vit D into the hsCRP model was evaluated by comparing the AUCs using the DeLong test. In addition, the incremental discriminative value of adverse-marker burden beyond a clinical model including age, sex, and endovascular therapy was assessed by comparing AUCs using the DeLong test. These DeLong comparisons were considered exploratory, and the reported *p* values were unadjusted. Sensitivity analyses included Firth penalized logistic regression, exclusion of patients with hsCRP >10 mg/L, additional covariate adjustment, continuous modeling of hsCRP and 25-OHD, and ordinal analysis of the full mRS scale. E-values were calculated to assess unmeasured confounding, and the minimum detectable effect size at 80% power was reported instead of observed power. All analyses were performed using R version 4.3.3, with a two-sided *p* < 0.05 considered statistically significant.

## 3. Results

The final cohort comprised 192 patients (Figure 1) distributed into low- (*n* = 76), moderate- (*n* = 57), and high- (*n* = 59) hsCRP-level groups. The baseline characteristics of the strata are summarized in Table 1. Two variables differed significantly among the groups. The mean serum 25-OHD levels exhibited a graded decline from the low- to the high-hsCRP group (21.2, 20.8, and 16.6 ng/mL, respectively; *p* = 0.032). White blood cell counts increased in parallel across groups (*p* = 0.043), consistent with hsCRP serving as an index of a broader cellular inflammatory response. Patients in the moderate- and high-hsCRP groups were numerically older than those in the low-hsCRP group, although the difference did not reach significance (*p* = 0.090). Endovascular therapy was numerically more frequent in the high-hsCRP group (27.1% versus 13.2% and 14.0%; *p* = 0.075). No other between-group differences reached significance.

Table 2 shows the correlation and regression findings for hsCRP levels. In the Spearman analysis, higher hsCRP levels were associated with higher (worse) NIHSS and mRS scores as well as lower (worse) BBS, MFT, K-MMSE, and K-MBI scores (all *p* < 0.05), demonstrating a consistent negative impact across all functional domains. For initial severity and unfavorable outcomes in BBS, MFT, K-MMSE, and mRS, the odds ratios (ORs) comparing the high- versus non-high-hsCRP groups were significant in the unadjusted Model 1 and remained significant in Model 2, which was adjusted for age and sex. Furthermore, these estimates changed little even in the adjusted Model 3, which additionally controlled for BMI, hypertension, diabetes, and, notably, Vit D status. After adjustment for endovascular therapy, the association with admission stroke severity was attenuated and no longer significant (OR, 2.13; 95% CI, 0.98–4.63; *p* = 0.057), whereas the remaining five outcomes remained significant after false discovery rate (FDR) correction (q ≤ 0.045).

The combined-abnormality group showed significantly higher odds of all six adverse outcomes compared with the reference group, with ORs ranging from 4.39 for poor balance to 12.54 for cognitive impairment (Table 3). All six associations remained significant after FDR correction (q < 0.01), and the comparison of the combined-abnormality group versus the reference group for poor mRS remained significant after Bonferroni correction (adjusted *p* = 0.035; Table S1). In contrast, the low-Vit D-only group showed significant associations with all six outcomes, but with smaller ORs (2.48–5.46), whereas the high-hsCRP-only group was significant only for poor manual dexterity. Firth penalized regression yielded slightly attenuated estimates but did not materially alter the findings.

No significant multiplicative interaction was observed for any of the six outcomes (all *p* ≥ 0.104). On the additive scale, significant interaction was observed only for cognitive impairment (RERI, 9.78; 95% CI, 1.73–58.65; AP, 0.78; 95% CI, 0.25–0.96; Table 4).

**Figure 2:**
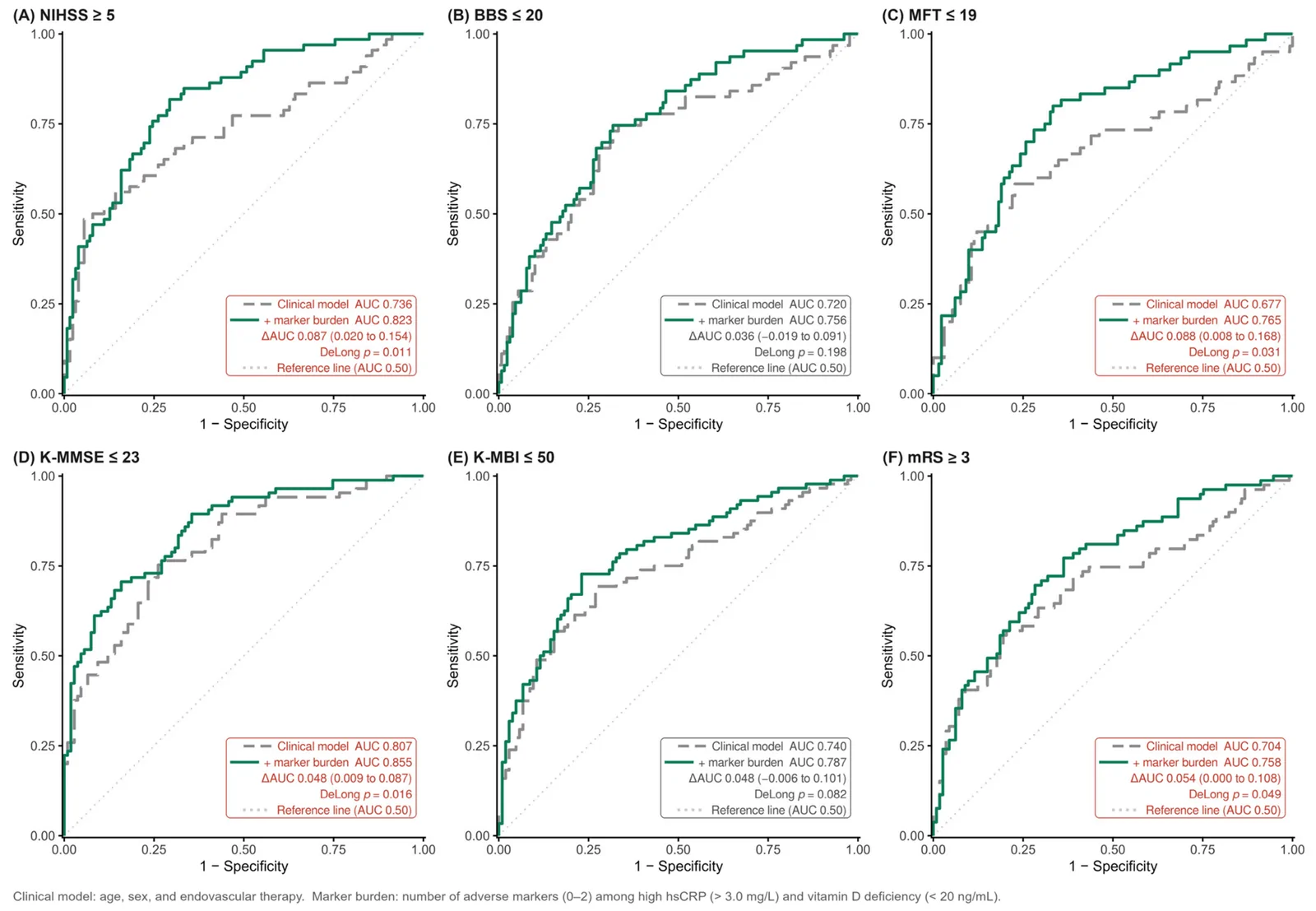
Comparison of discrimination between the clinical model and the clinical model plus adverse-marker burden. The clinical model included age, sex, and endovascular therapy. Adverse-marker burden was defined as the number of adverse markers (0–2): high hsCRP (>3.0 mg/L) and vitamin D deficiency (25-OHD < 20 ng/mL). AUCs were compared using the DeLong test. These DeLong comparisons were considered exploratory, and the reported *p* values were unadjusted. AUC, area under the receiver operating characteristic curve; hsCRP, high-sensitivity C-reactive protein; 25-OHD, 25-hydroxyvitamin D.

Adding Vit D to hsCRP alone modestly increased the AUC across the six outcomes, but none of the improvements reached statistical significance by the DeLong test (all *p* > 0.05; Table S2). In contrast, in exploratory analyses, adding the adverse-marker burden to a clinical model including age, sex, and endovascular therapy was associated with higher AUCs for four of the six outcomes, based on unadjusted DeLong *p* values: stroke severity (ΔAUC, 0.087; *p* = 0.011), motor function (ΔAUC, 0.088; *p* = 0.031), cognitive impairment (ΔAUC, 0.048; *p* = 0.016), and mRS (ΔAUC, 0.054; *p* = 0.049), whereas improvements for balance and activities of daily living were not significant (Figure 2).

The findings were robust across sensitivity analyses (Table S3). Exclusion of 21 patients with hsCRP >10 mg/L did not materially alter the associations, although the estimate for cognitive impairment showed the greatest attenuation (OR, 12.54 to 6.75). Additional adjustment for intravenous thrombolysis, season, or onset-to-admission interval also yielded consistent results. E-values were 3.73 (CI limit, 2.04) for poor functional outcome (mRS ≥ 3) and 6.54 (CI limit, 3.41) for cognitive impairment. Minimum detectable ORs at 80% power ranged from 3.26 to 4.02 and were lower than the observed estimates (Tables S4 and S5).

## 4. Discussion

In this cohort of 192 patients with acute ischemic stroke, an elevated hsCRP level at admission was associated with greater initial stroke severity and with poorer short-term recovery in balance, manual dexterity, cognition, and overall disability. The integration of Vit D status further strengthened this association; the subgroup with both high hsCRP and Vit D deficiency showed consistently elevated odds of unfavorable outcomes across the assessed domains, yielding adjusted odds that exceeded tenfold for severe admission stroke and poor cognitive recovery. Formal interaction testing provided no evidence of multiplicative interaction between high hsCRP and Vit D deficiency, whereas a significant additive interaction was observed only for cognitive impairment. Nevertheless, when the adverse-marker count was entered as an ordinal variable, a significant graded association was observed across all assessed outcomes. Taken together, these findings may be more consistent with a cumulative biomarker burden than with multiplicative amplification. Moreover, in exploratory analyses, adding the adverse-marker burden to a clinical model comprising age, sex, and endovascular therapy was associated with higher AUCs for four of the six outcomes, based on unadjusted DeLong *p* values. Clinically, this framework is attractive because a simple count of two inexpensive, routinely measured parameters separates low- from high-risk strata.

Recent Mendelian randomization evidence demonstrating that Vit D restrains CRP primarily within the deficiency range provides a specific mechanistic framework for this cumulative model [19]. In Vit D-replete patients, rising hsCRP levels occur against an intact anti-inflammatory background that blunts downstream secondary injury [17]. Conversely, this regulatory brake is absent in a deficient patient; thus, the same increase in hsCRP levels signifies an unopposed and amplified inflammatory load. This mechanism may explain why the dual-abnormality group ranked highest and why Vit D deficiency was a more consistent isolated predictor than hsCRP. Notably, although this mechanistic account suggests a potential interaction between the two markers, formal interaction testing showed no multiplicative interaction, whereas a significant additive interaction was observed only for cognitive impairment. Given its isolated nature and wide confidence intervals, this finding should be considered exploratory. 

Another important nuance was discrimination versus stratification. Although the combined hsCRP-plus-Vit D model achieved AUCs numerically exceeding that of a single marker, DeLong comparisons against hsCRP alone did not reach statistical significance (all *p* > 0.05). Although the combination did not measurably enhance continuous discrimination, in exploratory analyses, adding the adverse-marker burden to a clinical model comprising age, sex, and endovascular therapy was associated with higher AUCs for four of the six outcomes based on unadjusted DeLong *p* values. However, because the clinical model did not include admission NIHSS, these findings do not establish incremental prognostic value beyond established clinical predictors.

These results expand upon the existing literature linking inflammatory indices to stroke recovery. Our group previously demonstrated that a higher neutrophil-to-lymphocyte ratio predicts poorer short-term cognitive and daily living outcomes [6], whereas others have linked hsCRP to unfavorable recovery [10,18]. The present findings also move beyond our earlier report on Vit D in this cohort in three respects [20]. First, whereas the earlier report identified Vit D deficiency as a prognostic marker without accounting for its mechanistic basis, the present analysis shows that 25-OHD declines stepwise as hsCRP rises and evaluates their joint associations with clinical outcomes, thereby linking Vit D deficiency more explicitly to the inflammatory–nutritional axis rather than treating it as an isolated correlate. Second, and clinically more consequential, the earlier single-marker framing implicitly assigned comparable risk to all Vit D-deficient patients, whereas the present four-way classification shows that risk within the deficient group differs several-fold according to the inflammatory state; deficiency accompanied by high hsCRP levels conferred substantially greater odds of poor cognitive recovery (OR 12.54) than deficiency alone (OR 2.71). Third, the cumulative burden model yields a bedside metric—a count of 0–2 adverse markers—which a single-marker approach cannot provide. While Alfieri et al. observed that Vit D–deficient patients exhibited higher hsCRP and worse three-month mRS, their work evaluated the markers largely in parallel using a single disability endpoint [18]. The present study advances this concept by mapping the joint signal across multiple functional domains and demonstrating a generally graded association with increasing adverse-marker burden across all assessed outcomes.

From a translational and clinical standpoint, the simplicity of this cumulative burden framework is its greatest advantage. As both hsCRP and 25-OHD are routinely available upon admission without additional testing, patients presenting with both abnormalities may represent a readily identifiable subgroup at higher risk of unfavorable recovery and a potential target for closer monitoring and future intervention studies. Crucially, Vit D deficiency is a modifiable component within this inflammatory–nutritional axis; age, sex, and initial stroke severity cannot be altered, and hsCRP can be lowered only indirectly, whereas Vit D status can be corrected safely and inexpensively. This asymmetry provides a rationale for interventional trials examining whether early Vit D repletion in patients with acute stroke and Vit D deficiency attenuates systemic inflammation and enhances functional recovery on measures such as K-MBI and MFT. Our data further suggest how this trial might be designed: because the causal effect of Vit D on CRP appears to be confined to the deficiency range [19], and because the risk in this cohort was concentrated in the dual-abnormality stratum, supplementation trials enrolling unselected patients may dilute any true benefit. Therefore, the dual-abnormality subgroup identified here may represent a priority population for evaluation in future prospective intervention trials. Ultimately, this burden count was intended to supplement, rather than replace, established prognostic factors, such as age and admission NIHSS score, integrated into comprehensive clinical prediction models.

These findings have nutritional implications that extend beyond risk stratification. Vit D deficiency was strikingly common in this cohort, affecting 115 of 192 patients (59.9%), a prevalence consistent with global estimates for older and hospitalized populations [14]. It coincided with elevated hsCRP in 42 patients (21.9%), a nutritionally vulnerable, highly inflamed subgroup that neither measurement alone would reveal. The graded fall in 25-OHD across rising hsCRP strata observed here (21.2, 20.8, and 16.6 ng/mL) provides clinical corroboration, within acute stroke, of the inflammatory–nutritional coupling reported in other inflammatory conditions [17] and is causally supported by Mendelian randomization [19]. Notably, this axis extends to the musculoskeletal substrate on which rehabilitation depends. Higher hsCRP is associated with reduced muscle strength [12], whereas muscle mass deficit predicts worse stroke outcomes [13] and Vit D itself supports muscle function. Therefore, a patient with nutritional deficiency and inflammation may enter rehabilitation with both a heightened injury burden and a diminished capacity to respond to it, which is a plausible explanation for the pronounced deficits observed in manual dexterity and daily living function. Thus, Vit D status at admission emerges not only as a prognostic signal but also as a candidate nutritional target during the earliest phase of stroke care.

This study had certain limitations. First, of 339 patients meeting the eligibility criteria, 143 (42.2%) were excluded because three-week functional outcome data were unavailable, most commonly owing to early transfer or discharge before the scheduled assessment. Because such patients may include both the most severely affected and the mildest cases, the direction of the resulting selection bias cannot be determined a priori. Since baseline characteristics, including demographic characteristics, medical history and treatment-related variables, and laboratory findings, were not available for the excluded patients, a formal comparison between included and excluded patients could not be performed. Second, the dual-abnormality subgroup was relatively small (*n* = 42) and the isolated high-hsCRP subgroup smaller still (*n* = 17), producing wide confidence intervals; the magnitude of these estimates should therefore be interpreted with caution and validated in larger cohorts. In addition, although functional assessments were restricted to within three weeks of stroke onset to capture early outcomes, variation in the exact timing of assessment within this window may have influenced the measured functional status. Fourth, although patients receiving corticosteroid injections were excluded, the use of other anti-inflammatory or immunomodulatory medications was not specifically accounted for and may have influenced hsCRP levels, resulting in residual confounding. Furthermore, hsCRP was measured within 24 h of admission, but the interval from symptom onset to admission ranged up to 7 days (mean 2.2 ± 1.5 days). Because hsCRP follows a characteristic post-ischemic time course, variation in the onset-to-admission interval may have introduced measurement variability in hsCRP levels despite adjustment for this interval in sensitivity analyses. Finally, residual confounding from unmeasured factors such as stroke etiology (e.g., TOAST subtype), pre-stroke nutritional status, and infarct characteristics remains possible despite adjustment. Prospective multicenter studies incorporating serial biomarker sampling are warranted to validate these thresholds and their temporal dynamics.

## 5. Conclusions

Elevated hsCRP levels at admission were associated with greater initial stroke severity and poorer short-term recovery, mirroring the prognostic pattern previously established for Vit D deficiency. The integration of these two markers identified a distinct high-risk subgroup, with a generally graded increase in risk according to adverse-marker burden across all assessed outcomes. Since Vit D deficiency is a modifiable component of the inflammatory–nutritional axis, the dual-abnormality stratum identified here may represent a priority subgroup for evaluation in prospective trials of Vit D supplementation. Although adding Vit D to hsCRP alone did not significantly improve discrimination, exploratory analyses showed higher AUCs for four of the six outcomes when adverse-marker burden was added to a clinical model, based on unadjusted DeLong *p* values. These findings suggest that a simple burden count of routinely measured biomarkers may have potential for early risk assessment, although its incremental prognostic value requires prospective validation in more comprehensive clinical models.

## Figures and Tables

**Table 1:** Baseline characteristics of patients with acute ischemic stroke according to the level of hsCRP.

	Total (*n* = 192)	Low (*n* = 76)	Moderate (*n* = 57)	High (*n* = 59)	*p* Value
**Demographics**					
Age (years)	72.1 ± 12.9	69.6 ± 12.7	74.1 ± 10.9	73.3 ± 14.5	0.090
Female sex, *n* (%)	78 (40.6)	31 (40.8)	24 (42.1)	23 (39.0)	0.942
BMI (kg/m^2^)	24.0 ± 3.3	23.8 ± 3.1	23.5 ± 3.7	24.7 ± 3.3	0.118
**Medical history**					
Hypertension, *n* (%)	114 (59.4)	46 (60.5)	27 (47.4)	41 (69.5)	0.051
Diabetes mellitus, *n* (%)	69 (35.9)	30 (39.5)	18 (31.6)	21 (35.6)	0.642
Dyslipidemia, *n* (%)	71 (37.0)	31 (40.8)	18 (31.6)	22 (37.3)	0.552
Coronary artery disease, *n* (%)	24 (12.5)	6 (7.9)	12 (21.1)	6 (10.2)	0.061
Smoking, *n* (%)	65 (33.9)	28 (36.8)	22 (38.6)	15 (25.4)	0.253
Alcohol consumption, *n* (%)	67 (34.9)	27 (35.5)	16 (28.1)	24 (40.7)	0.359
Intravenous thrombolysis, *n* (%)	20 (10.4)	9 (11.8)	6 (10.5)	5 (8.5)	0.817
Endovascular therapy, *n* (%)	34 (17.7)	10 (13.2)	8 (14.0)	16 (27.1)	0.075
Length of hospital stay (days)	24 (19–36)	23 (20–31)	23 (18–35)	26 (19–45)	0.552
Onset to admission time (days)	2.2 ± 1.5	2.4 ± 1.6	2.0 ± 1.3	2.2 ± 1.7	0.352
**Season of blood sampling**					0.817
Spring, *n* (%)	33 (17.2)	11 (14.5)	10 (17.5)	12 (20.3)	
Summer, *n* (%)	72 (37.5)	29 (38.2)	23 (40.4)	20 (33.9)	
Autumn, *n* (%)	39 (20.3)	19 (25.0)	10 (17.5)	10 (16.9)	
Winter, *n* (%)	48 (25.0)	17 (22.4)	14 (24.6)	17 (28.8)	
**Laboratory findings**					
25-OHD (ng/mL)	19.7 ± 10.8	21.2 ± 10.6	20.8 ± 12.0	16.6 ± 9.4	0.032 *
Hemoglobin (g/dL)	13.7 ± 1.8	13.8 ± 1.7	13.6 ± 2.0	13.6 ± 1.8	0.725
Platelet (10^3^/uL)	222.7 ± 70.0	213.7 ± 59.7	231.5 ± 59.2	225.7 ± 89.1	0.325
WBC (10^3^/uL)	7.6 ± 2.9	6.9 ± 3.1	7.9 ± 2.5	8.1 ± 2.9	0.043 *
hsCRP (mg/L)	1.6 (0.6–4.8)	0.5 (0.4–0.7)	1.7 (1.3–2.2)	7.5 (5.2–13.1)	<0.001 ***
HbA1c (%)	6.2 ± 1.3	6.2 ± 1.1	6.2 ± 1.4	6.1 ± 1.5	0.848
TC (mg/dL)	180.0 ± 48.8	176.5 ± 46.4	186.6 ± 48.1	178.2 ± 52.6	0.474
TG (mg/dL)	141.2 ± 83.9	138.8 ± 78.6	146.2 ± 93.1	139.5 ± 82.5	0.866
HDL (mg/dL)	46.4 ± 11.9	48.2 ± 11.7	45.2 ± 9.8	45.2 ± 13.7	0.227
LDL (mg/dL)	105.4 ± 38.4	102.3 ± 37.3	109.8 ± 37.5	105.3 ± 40.8	0.539

* *p* < 0.05, *** *p* < 0.001. Continuous variables are presented as mean ± standard deviation, except hsCRP, which is shown as median (interquartile range) owing to its skewed distribution; categorical variables are given as *n* (%). Groups were compared by one-way analysis of variance, the Kruskal–Wallis test (hsCRP), or the chi-square test, as appropriate. 25-OHD, 25-hydroxyvitamin D; hsCRP, high-sensitivity C-reactive protein; WBC, white blood cell; HbA1c, hemoglobin A1c; TC, total cholesterol; TG, triglycerides; HDL/LDL, high-/low-density lipoprotein.

**Table 2:** Correlation and hierarchical logistic regression of serum hsCRP with admission severity and functional outcomes.

Outcome	Spearman ρ	Model 1 OR (95% CI)	Model 2 OR (95% CI)	Model 3 OR (95% CI)	Model 4 OR (95% CI)	q †
Moderate-to-severe stroke severity (NIHSS ≥ 5)	0.241 ***	2.51 (1.33–4.74) **	2.53 (1.32–4.86) **	2.31 (1.13–4.72) *	2.13 (0.98–4.63)	0.057
Poor BBS score (BBS ≤ 20)	−0.272 ***	2.53 (1.33–4.80) **	2.45 (1.26–4.77) **	2.38 (1.18–4.81) *	2.17 (1.05–4.49) *	0.045
Poor MFT score (MFT ≤ 19)	−0.247 ***	3.97 (2.06–7.65) ***	4.03 (2.07–7.87) ***	3.47 (1.71–7.03) ***	3.24 (1.57–6.69) **	0.009
Poor K-MMSE score (MMSE ≤ 23)	−0.31 ***	2.98 (1.58–5.62) ***	3.25 (1.58–6.67) **	3.00 (1.37–6.56) **	3.00 (1.28–7.05) *	0.023
Poor K-MBI score (MBI ≤ 50)	−0.349 ***	2.70 (1.44–5.09) **	2.80 (1.43–5.51) **	2.86 (1.37–5.99) **	2.74 (1.28–5.89) **	0.023
Poor mRS score (mRS ≥ 3)	0.172 *	2.40 (1.28–4.49) **	2.45 (1.27–4.70) **	2.45 (1.22–4.92) *	2.28 (1.10–4.71) *	0.040

* *p* < 0.05, ** *p* < 0.01, *** *p* < 0.001. Spearman ρ is the correlation of continuous hsCRP with the corresponding continuous score (NIHSS, BBS, MFT, K-MMSE, K-MBI, or mRS); a positive ρ links higher hsCRP to a higher score and a negative ρ to a lower score. Logistic models compare high (>3.0 mg/L) versus non-high hsCRP: Model 1, unadjusted; Model 2, + age and sex; Model 3, + BMI, hypertension, diabetes mellitus, and 25-OHD; Model 4, + endovascular therapy. † Benjamini–Hochberg false discovery rate across the six outcomes of Model 4. OR, odds ratio; CI, confidence interval; NIHSS, National Institutes of Health Stroke Scale; BBS, Berg Balance Scale; MFT, Manual Function Test; K-MMSE, Korean Mini Mental State Examination; K-MBI, Korean Modified Barthel Index; mRS, Modified Rankin Scale.

**Table 3:** Combined association of high hsCRP and Vit D deficiency (adjusted OR, 95% CI).

Outcome	High hsCRP Only (*n* = 17)	Low Vit D Only (*n* = 73)	Both (*n* = 42)	Both, Firth (95% CI) ‡	q †
Moderate-to-severe stroke severity (NIHSS >= 5)	1.67 (0.36–7.67)	5.46 (2.01–14.85) ***	10.35 (3.41–31.39) ***	9.23 (3.30–28.54)	<0.001
Poor BBS score (BBS <= 20)	2.43 (0.69–8.55)	2.49 (1.05–5.92) *	4.39 (1.67–11.59) **	4.15 (1.64–11.01)	0.006
Poor MFT score (MFT <= 19)	5.30 (1.47–19.16) *	3.02 (1.17–7.85) *	8.38 (3.00–23.43) ***	7.63 (2.93–21.80)	<0.001
Poor K-MMSE score (MMSE <= 23)	1.05 (0.26–4.26)	2.71 (1.14–6.44) *	12.54 (3.99–39.45) ***	11.08 (3.82–35.70)	<0.001
Poor K-MBI score (MBI <= 50)	2.66 (0.78–9.08)	3.58 (1.59–8.03) **	7.29 (2.73–19.46) ***	6.74 (2.66–18.18)	<0.001
Poor mRS score (mRS >= 3)	2.06 (0.61–6.97)	2.48 (1.12–5.49) *	4.64 (1.83–11.75) **	4.38 (1.80–11.14)	0.004

* *p* < 0.05, ** *p* < 0.01, *** *p* < 0.001. Reference, no abnormality (*n* = 60); high hsCRP, >3.0 mg/L; Vit D deficiency, 25-OHD < 20 ng/mL. Adjusted for age, sex and endovascular therapy. ‡ Firth penalized logistic regression with profile-likelihood confidence intervals, performed because the high-hsCRP-only stratum contains 17 patients; estimates were shrunk towards the null as expected but no inference changed. † Benjamini–Hochberg across the 18 tests of this table. hsCRP, high-sensitivity C-reactive protein; NIHSS, National Institutes of Health Stroke Scale; BBS, Berg Balance Scale; MFT, Manual Function Test; K-MMSE, Korean Mini Mental State Examination; K-MBI, Korean Modified Barthel Index; mRS, Modified Rankin Scale.

**Table 4:** Adverse-marker burden and formal interaction between high hsCRP and vitamin D deficiency.

Outcome	Burden, OR per +1 (95% CI)	*p*	q †	Multiplicative Interaction OR (95% CI)	*p*	RERI (95% CI)	AP (95% CI)
Moderate-to-severe stroke severity (NIHSS >= 5)	3.10 (1.83–5.26)	<0.001	<0.001	1.13 (0.19–6.61)	0.889	4.22 (−4.67 to 27.04)	0.41 (−0.67 to 0.79)
Poor BBS score (BBS <= 20)	2.09 (1.30–3.37)	0.003	0.003	0.73 (0.16–3.31)	0.681	0.48 (−5.78 to 7.61)	0.11 (−1.59 to 0.74)
Poor MFT score (MFT <= 19)	2.85 (1.73–4.69)	<0.001	<0.001	0.52 (0.11–2.41)	0.405	1.05 (−14.93 to 16.04)	0.13 (−1.69 to 0.72)
Poor K-MMSE score (MMSE <= 23)	3.27 (1.90–5.65)	<0.001	<0.001	4.43 (0.74–26.54)	0.104	9.78 (1.73 to 58.65)	0.78 (0.25 to 0.96)
Poor K-MBI score (MBI <= 50)	2.76 (1.70–4.49)	<0.001	<0.001	0.77 (0.17–3.50)	0.731	2.06 (−6.65 to 18.72)	0.28 (−1.15 to 0.78)
Poor mRS score (mRS >= 3)	2.17 (1.36–3.44)	0.001	0.001	0.91 (0.21–4.00)	0.899	1.10 (−4.43 to 9.39)	0.24 (−1.25 to 0.77)

All models adjusted for age, sex and endovascular therapy. Burden is the number of adverse markers (0–2) entered as an ordinal term. † Benjamini–Hochberg across the six trend tests. RERI and AP were estimated with percentile bootstrap confidence intervals (2000 resamples). Interaction analyses were considered exploratory and were excluded from the multiplicity adjustment. AP, attributable proportion; RERI, relative excess risk due to interaction.

## Data Availability

The de-identified data supporting the findings of this study are available from the corresponding author upon reasonable request.

## Supplementary Materials

The following supporting information can be downloaded at: https://www.mdpi.com/article/10.3390/nu18193244/s1, Table S1: Multiplicity adjustment; Table S2: Incremental discriminative value of adding 25-OHD to hsCRP; Table S3: Sensitivity analyses for the dual-abnormality group; Table S4: Minimum detectable effect sizes; Table S5: E-values for the dual-abnormality group.

## Abbreviations

The following abbreviations are used in this manuscript:

ANOVA	analysis of variance
AUC	area under the curve
BBS	Berg Balance Scale
hsCRP	High-sensitivity C-reactive protein
IL-6	interleukin-6
MBI	modified Barthel Index
MFT	Manual Function Test
mRS	modified Rankin Scale
NIHSS	National Institutes of Health Stroke Scale
OR	odds ratio
RERI	relative excess risk due to interaction
ROC	receiver operating characteristic
Vit D	vitamin D
25OHD	25-hydroxyvitamin D
