# Supplementary material for: Combined Association of High-Sensitivity C-Reactive Protein and Vitamin D with Short-Term Functional Outcomes in Acute Ischemic Stroke
Source: Nutrients. 2026 Oct 1;18(19):3244. doi: 10.3390/nu18193244 (PMC13666985; doi:10.3390/nu18193244)
Supplement: Supplementary file 1 [file nutrients-18-03244-s001.zip › nutrients-4516708-supplementary.pdf]

Table S1. Multiplicity adjustment

Family	Test	*p*	*q* (BH)	Bonferroni *p*
Table 3 (18 tests)	mRS ≥ 3 — Both	0.001	0.004	0.035
Table 3 (18 tests)	K-MMSE ≤ 23 — Both	<0.001	<0.001	<0.001
Table 3 (18 tests)	MFT ≤ 19 — Both	<0.001	<0.001	0.001
Table 3 (18 tests)	K-MBI ≤ 50 — Both	<0.001	<0.001	0.002
Table 3 (18 tests)	NIHSS ≥ 5 — Both	<0.001	<0.001	0.001
Table 3 (18 tests)	BBS ≤ 20 — Both	0.003	0.006	0.080
Table 2, Model 4 (6)	MFT ≤ 19	0.001	0.009	0.042
Table 2, Model 4 (6)	NIHSS ≥ 5	0.057	0.057	1.000
Table 4, trend (6)	All six outcomes	≤ 0.003	≤ 0.003	≤ 0.073

p values are unadjusted. *q* values were calculated using the Benjamini–Hochberg (BH) procedure within each test family. Bonferroni-adjusted *p* values are provided as a more conservative reference. The number of tests included in each multiplicity-adjustment family is shown in parentheses. NIHSS, National Institutes of Health Stroke Scale; BBS, Berg Balance Scale; MFT, Manual Function Test; K-MMSE, Korean Mini-Mental State Examination; K-MBI, Korean Modified Barthel Index; mRS, modified Rankin Scale; BH, Benjamini–Hochberg.

Table S2. Incremental discriminative value of adding 25-OHD to hsCRP

Outcome	AUC, hsCRP alone	AUC, hsCRP + 25-OHD	DeLong *p*
NIHSS ≥ 5	0.646	0.689	0.156
BBS ≤ 20	0.652	0.676	0.396
MFT ≤ 19	0.675	0.706	0.256
K-MMSE ≤ 23	0.673	0.720	0.072
K-MBI ≤ 50	0.669	0.706	0.158
mRS ≥ 3	0.639	0.669	0.281

NIHSS, National Institutes of Health Stroke Scale; BBS, Berg Balance Scale; MFT, Manual Function Test; K-MMSE, Korean Mini-Mental State Examination; K-MBI, Korean Modified Barthel Index; mRS, modified Rankin Scale; AUC, area under the receiver operating characteristic curve; hsCRP, high-sensitivity C-reactive protein; 25-OHD, 25-hydroxyvitamin D. Differences in AUC were evaluated using the DeLong test.

Table S3. Sensitivity analyses for the dual-abnormality group

Outcome	Main analysis	hsCRP > 10 excluded	Firth	+ IVT	+ season	+ onset
NIHSS ≥ 5	10.35 (3.41–31.39)	10.84 (3.09–38.09)	9.23 (3.30–28.54)	11.11 (3.56–34.67)	10.36 (3.37–31.80)	10.34 (3.40–31.40)
BBS ≤ 20	4.39 (1.67–11.59)	3.70 (1.22–11.28)	4.15 (1.64–11.01)	4.47 (1.68–11.89)	4.07 (1.52–10.85)	4.41 (1.67–11.63)
MFT ≤ 19	8.38 (3.00–23.43)	6.87 (2.18–21.61)	7.63 (2.93–21.80)	8.99 (3.15–25.65)	8.06 (2.86–22.73)	8.35 (2.98–23.39)
K-MMSE ≤ 23	12.54 (3.99–39.45)	6.75 (1.87–24.32)	11.08 (3.82–35.70)	14.23 (4.40–46.09)	13.30 (4.13–42.88)	12.68 (4.03–39.93)
K-MBI ≤ 50	7.29 (2.73–19.46)	7.15 (2.26–22.65)	6.74 (2.66–18.18)	7.08 (2.64–18.99)	7.01 (2.60–18.89)	7.28 (2.72–19.53)
mRS ≥ 3	4.64 (1.83–11.75)	3.49 (1.19–10.30)	4.38 (1.80–11.14)	4.35 (1.71–11.06)	4.14 (1.61–10.65)	4.61 (1.82–11.72)

All models adjusted for age, sex and endovascular therapy, plus the variable indicated. NIHSS, National Institutes of Health Stroke Scale; BBS, Berg Balance Scale; MFT, Manual Function Test; K-MMSE, Korean Mini-Mental State Examination; K-MBI, Korean Modified Barthel Index; mRS, modified Rankin Scale; hsCRP, high-sensitivity C-reactive protein; IVT, intravenous thrombolysis.

Table S4. Minimum detectable effect sizes

Outcome	Event rate in the reference group	Observed OR (Both)	Minimum detectable OR (80% power)
NIHSS ≥ 5	11.7%	10.35	4.02
BBS ≤ 20	16.7%	4.39	3.57
MFT ≤ 19	11.7%	8.38	4.02
K-MMSE ≤ 23	25.0%	12.54	3.26
K-MBI ≤ 50	23.3%	7.29	3.30
mRS ≥ 3	23.3%	4.64	3.30

OR, odds ratio; NIHSS, National Institutes of Health Stroke Scale; BBS, Berg Balance Scale; MFT, Manual Function Test; K-MMSE, Korean Mini-Mental State Examination; K-MBI, Korean Modified Barthel Index; mRS, modified Rankin Scale.

Table S5. E-values for the dual-abnormality group

Outcome	Adjusted OR (95% CI)	E-value, point estimate	E-value, CI limit
NIHSS ≥ 5	10.35 (3.41–31.39)	5.89	3.10
BBS ≤ 20	4.39 (1.67–11.59)	3.61	1.90
MFT ≤ 19	8.38 (3.00–23.43)	5.24	2.86
K-MMSE ≤ 23	12.54 (3.99–39.45)	6.54	3.41
K-MBI ≤ 50	7.29 (2.73–19.46)	4.84	2.69
mRS ≥ 3	4.64 (1.83–11.75)	3.73	2.04

Odds ratios were converted using the square-root approximation because outcome prevalence exceeded 15%. OR, odds ratio; CI, confidence interval; NIHSS, National Institutes of Health Stroke Scale; BBS, Berg Balance Scale; MFT, Manual Function Test; K-MMSE, Korean Mini-Mental State Examination; K-MBI, Korean Modified Barthel Index; mRS, modified Rankin Scale.
